# Comparative Hemostatic Effects of *Bothrops* spp. and *Daboia russelii* Venoms

**DOI:** 10.1155/bri/1756413

**Published:** 2026-06-30

**Authors:** Geovanna M. Malchias-Pires, Marcela Romanazzi, José R. Almeida, Jarred Williams, Pradeep Vijayakumar, Soheil Gilabadi, Rui Seabra Ferreira Júnior, Sakthivel Vaiyapuri, Manuela Berto Pucca

**Affiliations:** ^1^ Graduate Program in Bioscience and Biotechnology Applied to Pharmacy, School of Pharmaceutical Sciences, São Paulo State University (UNESP), Araraquara, São Paulo, Brazil, unesp.br; ^2^ School of Pharmacy, University of Reading, Reading, UK, reading.ac.uk; ^3^ Biomolecules Discovery Group, Universidad Regional Amazónica Ikiam, Tena, Napo, Ecuador; ^4^ Center for the Study of Venoms and Venomous Animals, São Paulo State University (UNESP), Botucatu, São Paulo, Brazil, unesp.br; ^5^ Department of Clinical Analysis, School of Pharmaceutical Sciences, São Paulo State University, Araraquara, São Paulo, Brazil, unesp.br

**Keywords:** *Bothrops*, coagulopathy, *Daboia russelii*, hemostasis, snake venoms, venom-induced consumption coagulopathy

## Abstract

Venoms from *Bothrops* species and *Daboia russelii* are among the leading causes of venom‐induced coagulopathy and snakebite‐related deaths. In this study, we comparatively evaluated the biochemical composition and hemostatic effects of venoms from seven *Bothrops* species and *D. russelii* using protein profiling and functional coagulation assays. Sodium dodecyl sulfate–polyacrylamide gel electrophoresis (SDS–PAGE) analysis revealed broadly similar molecular weight distributions between the two genera, consistent with the presence of snake venom metalloproteinases, serine proteases, and phospholipases A_2_, but with notable interspecific variability among *Bothrops* venoms. Enzymatic assays demonstrated heterogeneous metalloproteinase, serine protease, and phospholipase A_2_ activities across *Bothrops* species, whereas *D. russelii* venom showed strong phospholipase A_2_ activity and limited fibrinogenolytic activity. Functional coagulation analyses revealed marked differences between the genera. *Bothrops* venoms induced rapid clot initiation, with alterations in clot formation observed in a species‐dependent manner, accompanied by pronounced fibrinogen degradation, consistent with consumption coagulopathy driven by fibrinogenolysis and clot destabilization. In contrast, *D. russelii* venom exhibited a strong procoagulant profile, significantly reducing prothrombin time and activated partial thromboplastin time, with minimal direct fibrinogen degradation. Previous studies have shown that *D. russelii* venom also promotes rapid clot formation in rotational thromboelastometry assays. Together, these findings demonstrate that *Bothrops* spp. and *D. russelii* venoms converge functionally in disrupting hemostasis but do so through distinct biochemical strategies, providing experimental support for the divergent coagulation phenotypes observed clinically.

## 1. Introduction

The use of snake venoms as tools in therapeutic innovation and biotechnological research has attracted increasing scientific attention in recent decades [1–3]. These natural secretions represent complex biochemical arsenals, refined through evolution to efficiently interfere with critical physiological systems of prey or predators [4, 5]. Among the numerous biological effects induced by venom components, their actions on the hemostatic system are particularly relevant due to both their clinical implications and the mechanistic diversity involved [6, 7]. Many compounds isolated from venoms exhibit procoagulant, anticoagulant, fibrinolytic, or hemorrhagic activity, making them important models for studying hemostatic disorders and for developing innovative therapeutic agents [8–10]. Moreover, the investigation of these venoms has significantly contributed to the identification of novel molecular targets, including platelet receptors, coagulation factors, and components of the extracellular matrix [11, 12].

The genera *Bothrops* and *Daboia* belong to the family Viperidae, one of the most medically significant groups of venomous snakes worldwide [13]. However, they are classified into distinct subfamilies: *Bothrops* species are part of the subfamily Crotalinae (pit vipers), predominantly distributed in Central and South America, whereas *Daboia russelii*, commonly known as Russell’s viper, belongs to the subfamily Viperinae and is widely distributed across South and Southeast Asia [14–16]. These taxonomic and geographic distinctions are relevant, as they reflect differences in ecological niches, evolutionary pressures, and venom composition, which may ultimately influence their functional effects on hemostasis [17, 18].

Despite extensive independent characterization of *Bothrops* and *D.* venoms, direct comparative functional analyses integrating biochemical profiling and whole‐blood coagulation assays under standardized conditions remain limited [17, 19]. Most studies focus on individual species or isolated toxin activities, making cross‐genus functional comparisons difficult [20, 21]. A systematic side‐by‐side evaluation under identical experimental conditions is therefore necessary to clarify whether compositional similarities translate into comparable functional effects on hemostasis.

In this context, the present study aimed to perform a comparative biochemical and functional analysis of venoms from multiple *Bothrops* species and *D. russelii*, focusing on their effects on the human coagulation system. To this end, we employed protein profiling by sodium dodecyl sulfate–polyacrylamide gel electrophoresis (SDS–PAGE), enzymatic activity assays, fibrinogenolysis evaluation, rotational thromboelastometry (ROTEM), and conventional coagulation tests, including prothrombin time (PT) and activated partial thromboplastin time (aPTT). By systematically comparing their biochemical characteristics and functional effects under identical experimental conditions, this study provides experimental insights into distinct coagulation patterns induced by these medically relevant snake venoms, thereby contributing to a more integrated understanding of venom‐induced hemostatic modulation.

## 2. Materials and Methods

### 2.1. Venom Extraction

**FIGURE 1:**
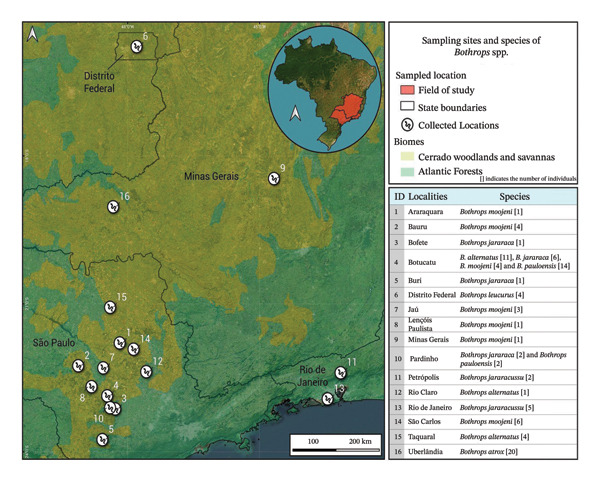
Map of the geographical distribution of the *Bothrops* species included in the study.

The venoms from the species *B. alternatus*, *B. atrox*, *B. jararaca*, *B. jararacussu*, *B. leucurus*, *B. moojeni*, and *B. pauloensis* were obtained from specimens maintained at the Serpentarium of the Center for the Study of Venoms and Venomous Animals of UNESP (CEVAP‐Botucatu‐SP, Brazil), under CRMV Registration No. 47038 and ART No. 05940/2024. The facility is registered with the Institutional Animal Care and Use Committee (CEUA‐IBTEC/UNESP; CNPJ 48.031.918/0001‐24) under License CIAEP No. 02.0472.2022 (issued on June 24, 2022), with authorization under Process No. SMA 000000002175/2013 and Wildlife Management Authorization No. 0000118253/2024. The specimens were collected from various municipalities in the states of São Paulo, Minas Gerais, and the Federal District, covering a wide geographical distribution and ensuring the biological representativeness of the samples analyzed (Figure 1). Each *Bothrops* venom corresponds to a single representative sample obtained from these collections. The venom of *D. russelii* was acquired from the Kentucky Reptile Zoo (Slade‐KY, USA). Differences in venom sourcing should be considered when interpreting interspecific comparisons.

### 2.2. SDS–PAGE

The venom composition was analyzed by SDS–PAGE under denaturing and reducing conditions. Protein separation was performed using a 15% resolving gel and a 5% stacking gel. Venom samples were prepared at a 1:1 ratio with sample buffer (50 mM Tris‐HCl, pH 6.8, 25% SDS, 10% glycerol, 0.002% bromophenol blue, and 5% 2‐mercaptoethanol). A total of 25 μg of venom protein per lane was loaded onto the gel. Electrophoresis was performed at a constant voltage of 100 V until adequate protein separation was achieved. Gels were stained with Coomassie Brilliant Blue R‐350 (PlusOne Coomassie Blue PhastGel, GE Healthcare, Chicago, IL, USA).

### 2.3. Enzymatic Assay

Enzymatic activities were evaluated using fluorogenic and chromogenic substrates to assess major toxin classes present in *Bothrops* venoms. All assays were performed using *Bothrops* spp. venoms as the experimental groups. Snake venom metalloproteinase activity was analyzed using DQ gelatin (ThermoFisher Scientific, Paisley, UK) as a fluorogenic substrate. Venoms (20 μg/mL) were mixed with 10 μg/mL of DQ gelatin in a total reaction volume of 100 μL (prepared in PBS) in a 96‐well microtiter plate. The plate was incubated at 37°C, and fluorescence was measured at various time points using an excitation wavelength of 485 nm and emission at 520 nm with a FLUOstar Omega microplate reader (BMG Labtech, Ortenberg, Germany). Similarly, serine protease activity was measured using Nα‐benzoyl‐L‐arginine 7‐amido‐4‐methylcoumarin HCl (Sigma‐Aldrich, Gillingham, Dorset, UK) as a fluorogenic substrate. After mixing the substrate (2 μM) with *Bothrops* venom samples, fluorescence was measured at an excitation wavelength of 366 nm and an emission wavelength of 460 nm at different time points. Phospholipase A_2_ (PLA_2_) activity was determined using the chromogenic substrate 4‐nitro‐3‐octanoyloxy benzoic acid (NOBA) in a reaction medium containing CaCl_2_. Substrate hydrolysis was monitored spectrophotometrically by measuring absorbance at 425 nm, according to previously described protocols. *Crotalus atrox* venom was used as a positive control for SVMP and SVSP assays. *D. russelii* venom was used as a positive control in the PLA_2_ assay due to its well‐characterized phospholipase activity and was not included as a comparative experimental sample [22].

### 2.4. Human Blood Collection and Plasma Preparation

Blood samples from four healthy human volunteers were collected according to procedures approved by the Research Ethics Committee of the University of Reading (UREC 17/17). After written informed consent, blood was collected by venipuncture into vacutainers containing 3.2% (w/v) sodium citrate. For plasma preparation, whole blood was centrifuged at 1400 × g for 10 min at 20°C. The upper transparent layer of plasma was carefully collected and used in coagulation experiments. Citrated whole blood was used directly in ROTEM experiments without further processing.

### 2.5. Hemodynamic Assays

It is important to note that not all assays were performed under fully equivalent experimental conditions for *Bothrops* spp. and *D. russelii* venoms. Some assays were specifically designed to investigate distinct mechanistic aspects of venom activity or were limited by experimental constraints. Consequently, direct comparisons between genera were only performed in experiments where conditions were fully comparable and methodologically appropriate.

#### 2.5.1. ROTEM

ROTEM analysis was performed using a ROTEM delta instrument (Werfen, Warrington, UK) to determine the effects of *Bothrops* spp. venoms on various parameters of whole blood coagulation. The INTEM and EXTEM assays were carried out to assess the venoms’ effects on the intrinsic and extrinsic (as well as common) coagulation pathways, respectively. For each assay, 20 μg/mL of *Bothrops* venom was mixed with 300 μL of citrated human whole blood and predefined volumes of the respective reagents according to the manufacturer’s instructions. For both INTEM and EXTEM assays, blood samples were recalcified using a STARTEM reagent (0.2 M CaCl_2_ in HEPES buffer, pH 7.4). Coagulation was initiated using intrinsic activators (ellagic acid and phospholipids) or extrinsic activators (recombinant tissue factor and phospholipids), depending on the assay. Clot formation and lysis were monitored for 60 min.

ROTEM assays were performed exclusively with *Bothrops* venoms. Data regarding *D. russelii* were not generated in this study and are referenced from previously published work [23] for comparative and contextual purposes.

#### 2.5.2. PT and aPTT

PT and aPTT were measured according to the manufacturer’s instructions using an automated coagulation analyzer (Ceveron T100, Technoclone, Vienna, Austria). Venom (20 μg/mL) was incubated with plasma obtained from healthy donors prior to analysis. The PT reagent (thromboplastin with 25 mM CaCl_2_) and the aPTT reagent (phospholipid/silica with 25 mM CaCl_2_) were supplied by the manufacturer. Clotting times were automatically recorded by the Ceveron T100 system.

#### 2.5.3. Fibrinogenolytic Assay

The fibrinogenolytic activity of the venoms was tested using a modification of the protocol described by Menezes et al. [24]. Fifty micrograms of human fibrinogen (MP Biomedicals, Santa Ana, CA, USA) was incubated with 20 μg/mL of each individual venom from *Bothrops* spp. and *D. russelii* in 0.05 M Tris‐HCl buffer, pH 8.0, at 37°C. Reactions were carried out at different time points (0, 1, 3, 6, and 24 h). At each time point, the reaction was stopped by the addition of a reducing sample buffer for SDS–PAGE electrophoresis (50 mM Tris‐HCl, pH 6.8, 25% SDS, 10% glycerol, 0.002% bromophenol blue, and 5% 2‐mercaptoethanol).

Fibrinogenolytic activity was demonstrated by polyacrylamide gel electrophoresis (12.5% SDS–PAGE), as described by Laemmli [25]. The gels were stained with Coomassie Brilliant Blue R‐250, and Bio‐Rad molecular weight standards (Bio‐Rad Laboratories, Cat. No. 161‐0374, Hercules, CA, USA) were used to estimate the masses of the protein bands.

### 2.6. Statistical Analysis

Statistical analyses were performed using GraphPad Prism 11.0.0 (GraphPad Software Inc., USA). Data are presented as mean ± SD from four independent human donors (biological replicates, *n* = 4), each exposed to all experimental conditions (repeated‐measures design). Analyses were conducted using donor‐level data without technical replicates. Interspecies comparisons are based on a single pooled venom per species and should be interpreted with caution. Differences among conditions were evaluated using one‐way repeated‐measures ANOVA, followed by Dunnett’s test to compare treated groups with the control. Statistical significance was set at *p* < 0.05.

## 3. Results

### 3.1. *Bothrops* spp. and Russell’s Viper Venoms Show Comparable Electrophoretic Profiles

**FIGURE 2:**
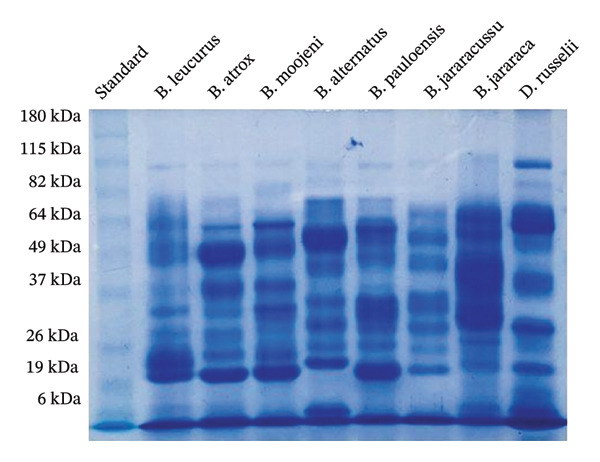
Electrophoretic profiles of crude snake venoms from *Bothrops* species (*B. leucurus*, *B. atrox*, *B. moojeni*, *B. alternatus*, *B. pauloensis*, *B. jararaca*, and *B. jararacussu*) and *D. russelii*, analyzed by 15% SDS–PAGE. Molecular weight standards were determined using a BLUeye prestained protein ladder (Sigma‐Aldrich, Cat. No. 94964). Samples were loaded at 25 μg per lane and visualized by Coomassie Brilliant Blue staining.

The electrophoretic patterns of the crude venoms from *B. jararaca*, *B. jararacussu*, *B. alternatus*, *B. pauloensis*, *B. atrox*, *B. moojeni*, *B. leucurus*, and *D. russelii* revealed complex band distributions spanning a broad range of molecular weights (Figure 2). Protein bands were observed within molecular weight ranges typically associated with snake venom metalloproteinases (approximately 30–100 kDa), serine proteases (approximately 25–35 kDa), and PLA_2_ (approximately 15–20 kDa), consistent with previously reported profiles of viperid venoms [26].

The venom of *D. russelii* also exhibited bands within these molecular weight intervals. As SDS–PAGE provides qualitative information, differences in the band intensity were not used to infer relative protein abundance.

### 3.2. *Bothrops* spp. Venoms Exhibit Differing Abilities to Hydrolyze Synthetic Substrates

**FIGURE 3:**
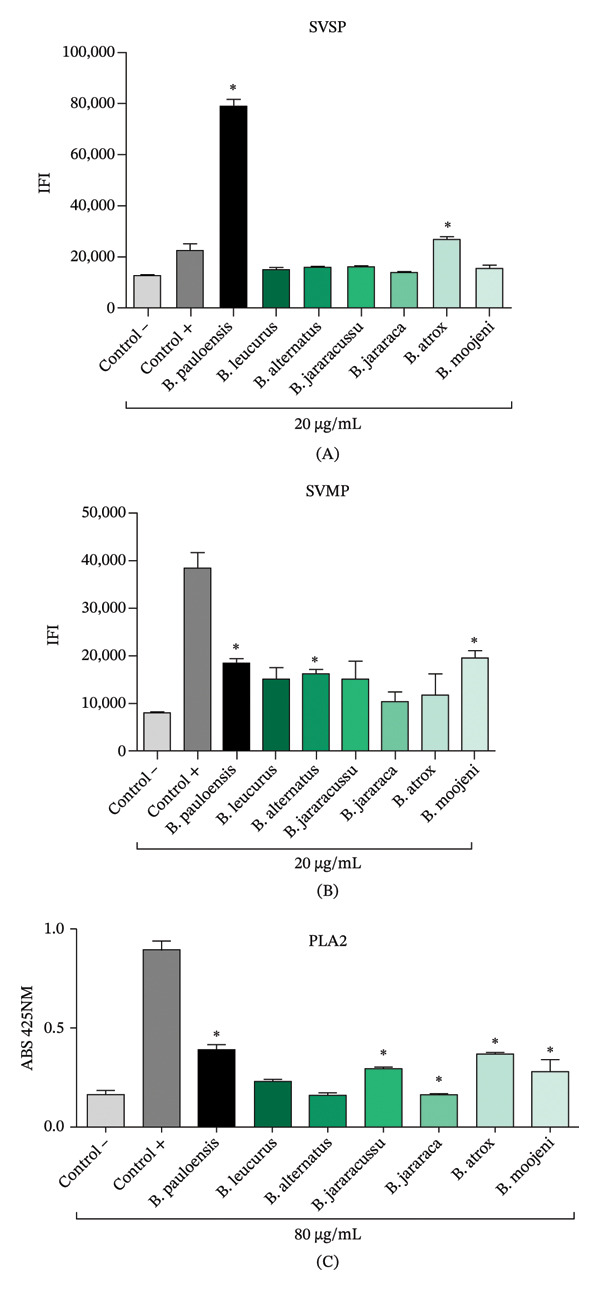
Enzymatic activities of *Bothrops* spp. venoms. The activities of SVSP (A), SVMP (B), and PLA_2_ (C) were measured using specific fluorogenic or chromogenic substrates. Baseline fluorescence obtained from the negative control (NC; substrate in the absence of venom) at 90 min was set as 100% to calculate enzymatic activities in venom samples at the same time point. *Crotalus atrox* venom (20 μg/mL) was used as a positive control (PC) in the SVSP and SVMP assays. *Daboia russelii* venom (80 μg/mL) was used as a positive control (PC) in the PLA_2_ assay. Abbreviations: SVSP, snake venom serine proteases; SVMP, snake venom metalloproteinases; PLA_2_, phospholipase A_2_.

The enzymatic assays revealed distinct catalytic profiles among *Bothrops* venoms (Figure 3). Importantly, the detection of protein bands in SDS–PAGE does not necessarily imply measurable catalytic activity under the assay conditions employed.

In the snake venom serine protease (SVSP) assay (Figure 3A), statistically significant activity was observed only for *B. pauloensis* and *B. atrox*, whereas the remaining venoms did not differ significantly from the negative control. In the snake venom metalloproteinase (SVMP) assay (Figure 3B), significant activity was detected in *B. pauloensis*, *B. alternatus*, and *B. moojeni.* The other venoms showed detectable fluorescence signals but did not reach statistical significance relative to the negative control.

Regarding PLA_2_ activity (Figure 3C), statistically significant activity was observed in *B. pauloensis*, *B. jararacussu*, *B. atrox*, and *B. moojeni*, while the remaining venoms did not differ significantly from control values.

It is important to note that *D. russelii* venom was not included as a comparative experimental group in these assays, but was selectively used as a positive control in the PLA_2_ assay.

Collectively, these results indicate that although proteins compatible with major toxin families were observed in electrophoretic profiles, catalytic activity varied among species and was not uniformly detected under the experimental conditions applied.

### 3.3. Differential Fibrinogen Degradation Profiles of *Bothrops* spp. and *D. russelii* Venoms

**FIGURE 4:**
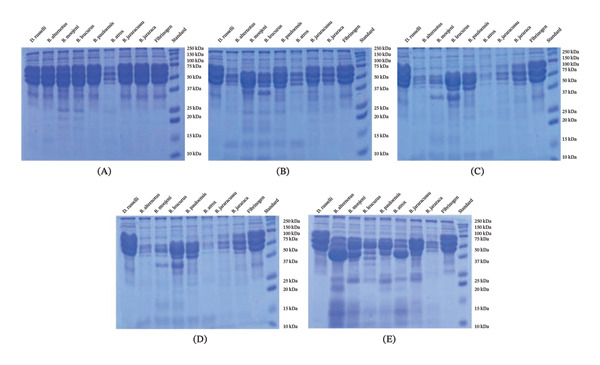
Analysis of the fibrinogenolytic activity of snake venoms from the *Bothrops* genus and the *D. russelii*. Each panel (A–E) represents a distinct incubation time: (A) 0 h, (B) 1 h, (C) 3 h, (D) 6 h, and (E) 24 h. The following samples were analyzed: *D. russelii*, *B. alternatus*, *B. moojeni*, *B. leucurus*, *B. pauloensis*, *B. atrox*, *B. jararacussu*, *B. jararaca*, human fibrinogen (control), and molecular weight marker (standard).

The fibrinogenolytic assay evaluated the degradation of human fibrinogen after incubation with crude venoms from *Bothrops* spp. and *D. russelii*. Samples were analyzed by 12% SDS–PAGE under denaturing conditions, and the degradation of bands corresponding to the fibrinogen α (∼66 kDa), β (∼52 kDa), and γ (∼46.5 kDa) chains was visually assessed over time. Figure 4 shows the proteolytic profile of fibrinogen incubated with venoms for 0, 1, 3, 6, and 24 h. At the 0‐h time point (Figure 4A), *Bothrops atrox* venom showed early fibrinogen degradation, evidenced by weakening of bands within the 40–75 kDa range in the corresponding lane. After 1 h of incubation (Figure 4B), additional venoms demonstrated visible degradation of the fibrinogen *α* chain, including *B. alternatus*, *B. leucurus*, *B. atrox*, and *B. jararaca*. The venoms of *B. moojeni* and *B. jararacussu* showed noticeable fibrinogen degradation after 3 h of incubation, characterized by the reduction of the *α* chain followed by progressive weakening of β and γ chains.

In contrast, *D. russelii* and *B. pauloensis* maintained fibrinogen banding patterns comparable to the fibrinogen control during the evaluated time points, suggesting limited fibrinogenolytic activity under the experimental conditions tested.

### 3.4. ROTEM‐Based Assessment of Coagulation Changes Induced by *Bothrops* spp. Venoms

**FIGURE 5:**
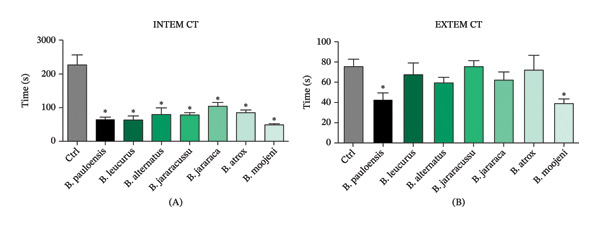
Effect of *Bothrops* spp. venoms on ROTEM parameters. Crude venoms (20 μg/mL) were evaluated using citrated human whole blood in INTEM and EXTEM assays. Clot formation was monitored for 60 min using a ROTEM delta instrument. Coagulation time (CT) is shown in panels (A) (INTEM) and (B) (EXTEM). Data are presented as mean ± standard deviation (*n* = 4 donors). Statistical analysis was performed using repeated‐measures one‐way ANOVA followed by Dunnett’s multiple comparisons test. *p* < 0.05 was considered statistically significant compared to the control (GraphPad Prism).

**FIGURE 6:**
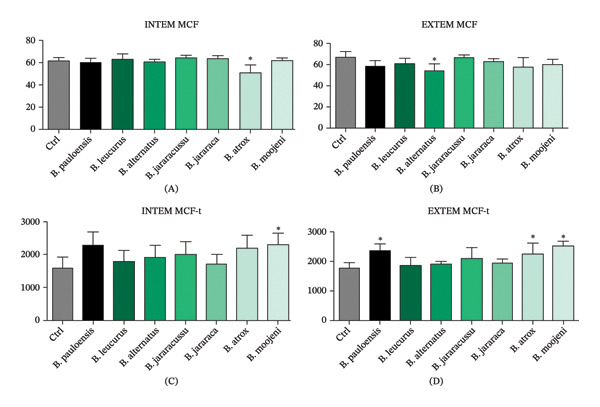
Effect of *Bothrops* spp. venoms on ROTEM parameters. Crude venoms (20 μg/mL) were evaluated using citrated human whole blood. Analyses were conducted using INTEM and EXTEM reagents, and clot formation was monitored for 60 min on a ROTEM delta instrument. The graphs show maximum clot firmness (MCF) (A, C) and time to maximum clot firmness (MCF‐t) (B, D). Data are presented as mean ± standard deviation (*n* = 4 donors). Statistical analysis was performed using repeated‐measures one‐way ANOVA followed by Dunnett’s multiple comparisons test. *p* < 0.05 was considered statistically significant compared to the control group (GraphPad Prism).

To examine in detail the effects of *Bothrops* venoms on blood coagulation, ROTEM analysis was performed using citrated human whole blood. The EXTEM analysis (which assesses the extrinsic and common pathways of the coagulation cascade) demonstrated accelerated coagulation (CT‐time from the start of the test to the clot reaching 2 mm amplitude) for *Bothrops* venoms (20 μg/mL) (Figures 5 and 6) compared to the control. The INTEM analysis (which assesses the intrinsic and common pathways of the coagulation cascade) also revealed changes in CT (accelerated coagulation) and MCF‐t of the intrinsic pathway (Figures 5 and 6). CT was the most consistent parameter affected, showing a reduction in coagulation time in blood from all four donors for venoms from all seven *Bothrops* species when compared to the control.

For MCF, only *B. alternatus* in the extrinsic pathway showed a statistically significant difference compared to the control, demonstrating faster achievement of maximum clot firmness (MCF) relative to the control and other *Bothrops* venoms. No consistent reduction in clot firmness was observed across the remaining species. The MCF‐t of the intrinsic pathway showed the longest time to reach MCF for *B. moojeni*, while in the extrinsic pathway, the time was longer for *B. pauloensis*, *B. moojeni*, and *B. atrox.*

Data regarding *D. russelii* in ROTEM assays were not generated in the present study and are therefore not included in this analysis.

The venoms of *Bothrops* showed reduced clotting times, indicating accelerated clot initiation. However, changes in clot firmness were not consistently observed across all species. A statistically significant difference in MCF was detected only for *B. alternatus* in the extrinsic pathway. These findings suggest that, although clot initiation is accelerated, alterations in clot stability may occur in a species‐dependent manner, consistent with a consumptive coagulopathy profile.

### 3.5. Differential Effects of *Bothrops* spp. and *D. russelii* Venoms on Extrinsic and Intrinsic Coagulation Pathways

**FIGURE 7:**
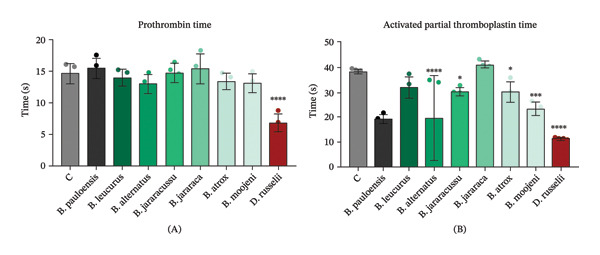
PT and aPTT alterations induced by *Bothrops* spp. and *D. russelii* venoms. Venoms (20 μg/mL) were incubated with plasma and analyzed using PT (A) and aPTT (B) reagents on a Ceveron T100 coagulation analyzer. Data are presented as mean ± SD (*n* = 4 donors). Statistical analysis was performed using repeated‐measures one‐way ANOVA followed by Dunnett’s multiple comparisons test. *p* < 0.05 was considered statistically significant compared to the control group (GraphPad Prism).

The effects of *Bothrops* spp. and *D. russelii* venoms on plasma coagulation were evaluated using PT and aPTT assays (Figure 7). In the PT assay (Figure 7A), most *Bothrops* venoms produced no significant change in clotting time compared with the control, indicating a limited effect on the extrinsic pathway under the conditions tested. In contrast, *D. russelii* venom caused a marked and significant reduction in PT, consistent with a strong procoagulant effect mediated through the extrinsic and/or common coagulation pathways.

In the aPTT assay (Figure 7B), several *Bothrops* venoms significantly shortened clotting times relative to the control, although the magnitude of this effect varied among species. Notably, *B. pauloensis*, *B. jararacussu*, *B. moojeni*, and *B. atrox* induced significant reductions in aPTT, whereas other *Bothrops* venoms showed more moderate or variable effects. *D. russelii* venom produced the most pronounced reduction in aPTT, significantly shortening clotting time compared with both the control and Bothrops venoms. Overall, these results indicate that *D. russelii* venom exerts a potent and consistent procoagulant effect on both PT and aPTT, whereas *Bothrops* venoms primarily affect the intrinsic pathway, displaying interspecific variability in their impact on plasma coagulation.

## 4. Discussion

This study provides a controlled functional comparison between *Bothrops* spp. and *D. russelii* venoms using integrated biochemical assays and viscoelastic coagulation testing. Although SDS–PAGE profiles revealed protein bands within molecular weight ranges consistent with major toxin families such as SVMPs, SVSPs, and PLA_2_s across species, enzymatic activity was not uniformly detected among *Bothrops* venoms. Only a subset of venoms exhibited statistically significant catalytic activity in substrate‐based assays, demonstrating that compositional similarity does not necessarily translate into equivalent functional potency. This lack of direct correlation highlights the limitations of inferring biological activity solely from electrophoretic or compositional data and underscores the importance of experimental functional validation [27, 28].

Fibrinogenolytic assays revealed marked interspecific differences in degradation kinetics. Some venoms promoted rapid cleavage of the fibrinogen α chain, followed by progressive β and γ chain degradation, whereas others induced delayed or limited proteolysis under identical conditions. These findings are consistent with the recognized multifactorial nature of venom‐induced consumption coagulopathy (VICC), in which multiple enzymatic components act simultaneously on coagulation substrates [29, 30].

Evaluation of whole‐blood coagulation using ROTEM further emphasized the complexity of venom‐induced hemostatic modulation. Alterations in coagulation time and clot firmness were species‐dependent and did not consistently parallel the magnitude of isolated enzymatic activity. In particular, while accelerated clot initiation was consistently observed, significant alterations in clot firmness were restricted to specific cases (e.g., *B. alternatus* in the extrinsic pathway). Viscoelastic assays capture the functional balance between procoagulant and anticoagulant components acting simultaneously in whole blood, providing an integrated assessment of clot formation that cannot be predicted from substrate‐specific assays alone. The absence of direct concordance between enzymatic profiles and clot formation parameters illustrates the multifactorial nature of venom‐induced coagulopathy [31, 32].

Similarly, PT and aPTT assays demonstrated heterogeneous interference with the extrinsic and intrinsic pathways, reinforcing that venoms sharing common toxin families may nonetheless exert distinct pathway‐specific effects. Although both *Bothrops* spp. and *D. russelii* are characterized by venoms rich in metalloproteinases, serine proteases, and PLA_2_, our results indicate that compositional convergence does not imply mechanistic equivalence. Notably, while ROTEM analyses in the present study were performed exclusively with *Bothrops* venoms, previous studies have reported that *D. russelii* venom induces rapid and consistent procoagulant responses in viscoelastic assays.

Overall, our data demonstrate that despite overlapping toxin families, the hemostatic effects of *Bothrops* spp. and *D. russelii* venoms are species‐specific and shaped by differences in enzymatic efficiency, relative abundance, and interactions among components within crude venom. These findings reinforce that comprehensive venom characterization requires the integration of structural and functional approaches, as protein profiling alone is insufficient to predict biological impact on coagulation.

Although the venom of *D. russelii* exhibits procoagulant activity in vitro, it is important to interpret this finding within the clinical context of envenoming. The procoagulant effects observed in experimental assays reflect early activation of the coagulation cascade, which, in vivo, contributes to the development of VICC. This condition is characterized by the progressive depletion of clotting factors, frequently leading to incoagulable blood, systemic bleeding, thrombocytopenia, and, in severe cases, organ damage. Therefore, the apparent procoagulant activity does not translate into a hypercoagulable state in patients, but rather into a consumptive coagulopathy with hemorrhagic manifestations.

## 5. Conclusions

This study shows that *Bothrops* spp. and *D. russelii* venoms disrupt human coagulation through functionally convergent but mechanistically distinct strategies. While both genera share major toxin families, *Bothrops* venoms exhibited interspecific variability and pronounced fibrinogen degradation, with alterations in clot formation observed in a species‐dependent manner. In contrast, *D. russelii* venom has been reported to produce a more uniform and potent procoagulant effect, characterized by rapid clot initiation and significant shortening of PT and aPTT, with minimal direct fibrinogenolysis. These findings demonstrate that similar coagulopathic outcomes can arise from different biochemical strategies and highlight the importance of genus‐specific functional characterization when investigating venom‐induced hemostatic disturbances.

## Author Contributions

Conceptualization, Geovanna M. Malchias‐Pires and Marcela Romanazzi; methodology, Manuela Berto Pucca and Sakthivel Vaiyapuri; software, Geovanna M. Malchias‐Pires, Marcela Romanazzi, José R. Almeida, and Jarred Williams; validation, Geovanna M. Malchias‐Pires, Marcela Romanazzi, José R. Almeida, and Jarred Williams; formal analysis, Geovanna M. Malchias‐Pires, Marcela Romanazzi, José R. Almeida, and Jarred Williams; investigation, Geovanna M. Malchias‐Pires and Marcela Romanazzi; resources, Sakthivel Vaiyapuri; data curation, Geovanna M. Malchias‐Pires, Marcela Romanazzi, José R. Almeida, and Jarred Williams; writing–original draft preparation, Geovanna M. Malchias‐Pires, Marcela Romanazzi, and José R. Almeida; writing–review and editing, Geovanna M. Malchias‐Pires, Marcela Romanazzi, José R. Almeida, Manuela Berto Pucca, and Sakthivel Vaiyapuri; visualization, Manuela Berto Pucca and Sakthivel Vaiyapuri; supervision, Manuela Berto Pucca and Sakthivel Vaiyapuri; project administration, Manuela Berto Pucca and Sakthivel Vaiyapuri; funding acquisition, Geovanna M. Malchias‐Pires, Marcela Romanazzi, and José R. Almeida.

## Funding

Manuela Berto Pucca was supported by the Brazilian National Council for Scientific and Technological Development (CNPq) through a research fellowship (Grant No. 305778/2023‐4). Geovanna M. Malchias‐Pires and Marcela Romanazzi received FAPESP scholarships (Geovanna M. Malchias‐Pires: Grant Nos. 2023/15381‐1 and 2024/08990‐4; Marcela Romanazzi: Grant No. 2024/01801‐1 and 2025/11949‐9). This study was also financed in part by the Coordenação de Aperfeiçoamento de Pessoal de Nıvel Superior‐Brasil (CAPES) ‐ Finance Code 001.

## Disclosure

All authors have read and agreed to the published version of the manuscript. The funding agencies were not involved in study design, data collection and analysis, decision to publish, or preparation of the manuscript.

## Ethics Statement

The study was approved by the Research Ethics Committee of the University of Reading (Protocol Code UREC 17/17).

## Consent

Written informed consent was obtained from all healthy volunteers involved in this study.

## Conflicts of Interest

The authors declare no conflicts of interest.

## Data Availability

The data supporting the findings of this study are available from the corresponding author upon reasonable request.
